# Gitelman Syndrome Manifesting With Acute Hypokalemic Paralysis: A Case Report

**DOI:** 10.7759/cureus.45997

**Published:** 2023-09-26

**Authors:** Rahul Gunde, Jayashankar CA, Nuthan Bhat, Vivek Bhat, Suresha Kodapala

**Affiliations:** 1 Neurology, Vydehi Institute of Medical Sciences and Research Centre, Bangalore, IND; 2 Internal Medicine, Vydehi Institute of Medical Sciences and Research Centre, Bangalore, IND; 3 Internal Medicine, St. John's Medical College, Bangalore, IND

**Keywords:** guillain-barré syndrome (gbs), rhabdomyolysis, acute hypokalemic paralysis, familial hypokalemia-hypomagnesemia, gitelman syndrome

## Abstract

Gitelman syndrome (GS) is a rare renal tubulopathy, classically characterized by renal salt wasting and metabolic alkalosis. It is usually an incidental diagnosis, being asymptomatic or with mild symptoms. GS manifesting with acute flaccid paralysis is extremely uncommon. We report a case of GS that mimicked Guillain-Barré syndrome (GBS), manifesting with acute hypokalemic paralysis. A middle-aged male with no known comorbidities presented to our center with paresthesias of all four limbs for one month and progressive, asymmetric limb weakness over the past eight days. Neurological examination revealed hypotonia, global areflexia, and power ranging from 3/5 to 4/5 in all four limbs, leading to our initial clinical diagnosis of GBS. Our patient’s laboratory panel revealed hypokalemia, hypomagnesemia, metabolic alkalosis, and hypocalcemia, characteristic of GS. Additionally, he had significantly elevated creatine phosphokinase, suggestive of rhabdomyolysis. Further urine studies revealed renal potassium wasting, confirming the diagnosis of GS. Whole exome genome sequencing for common causative genes and workup for autoimmune disease were both negative. With gradual electrolyte correction, the patient rapidly improved symptomatically. Our case highlights an uncommon initial presentation of GS and emphasizes the need for more literature on its manifestations from the Indian subcontinent.

## Introduction

Gitelman syndrome (GS) is a rare, salt-losing tubulopathy characterized by hypokalemia, metabolic alkalosis, hypomagnesemia, and hypocalciuria [1]. It is often asymptomatic or presents with mild symptoms such as fatigue, myalgia, muscle cramps, salt craving, and excessive thirst [1]. Acute flaccid paralysis as the initial manifestation of GS is incredibly rare [2-6]. We present a case of a middle-aged male patient with Gitelman syndrome presenting with acute progressive quadriparesis.

Aspects of this work were presented at the Indian Academy of Neurology Annual Conference on September 17, 2023.

## Case presentation

A 59-year-old male from West Bengal, India, with no other comorbidities, presented to our tertiary care center with complaints of diffuse paresthesia of his upper and lower limbs for one month and progressive weakness of all four limbs over the last eight days. He had weakness of the left upper limb for eight days, which had both proximal aspects in the form of difficulty in lifting his arm overhead and distal aspects in the form of inability to button and unbutton his shirt. He also had weakness of the right lower limb that progressed from an initial change in gait to an inability to lift his leg over the past eight days. Over the past two days, he has had weakness of the right upper limb in the form of difficulty in lifting the arm above his shoulder and difficulty gripping objects. He has had weakness in the left lower limb for one day. The weakness progressed in an asymmetric fashion. Over the last three days, there was a relatively rapid progression, from requiring support to walk to being wheelchair-bound. He also reported recurrent spasms of all four limbs. He had a history of occasional episodes of diuresis over the past month. He denied urinary or bowel incontinence, prior similar episodes of weakness, or significant family history. He also denied any other systemic symptoms, the use of supplements, special diets, or surreptitious intake of medications causing dyselectrolytemia such as insulin, chloroquine, thiazide, or loop diuretics, among others. There was no significant history of exposure to sick contacts and animal contact or travel history.

On examination of vital parameters, he had a temperature of 98.8°F, pulse rate of 80 beats/minute, blood pressure of 128/82 mmHg, and respiratory rate of 16 cycles/minute. He was alert and oriented and had no cranial nerve abnormalities. He had a positive Chvostek’s sign and occasional involuntary spasms of his hands. Motor examination revealed hypotonia of all four limbs and absent bilateral deep tendon reflexes including the biceps, brachioradialis, triceps, patellar, and ankle reflexes. Power ranged from 3/5 to 4/5 on the Medical Research Council (MRC) scale. The bilateral shoulder, hip, knee, and ankle had a power of 3/5. The right elbow and wrist had a power of 4/5, whereas the left elbow and wrist had a power of 3/5. The right-hand grip was stronger at 70%, whereas the left-hand grip was 50%. The sensory system examination including touch, pain, temperature, proprioception, and cortical sensation was within normal limits. There was no sphincter involvement, meningeal signs, or spinal abnormalities. He did not have signs of respiratory insufficiency.

He was admitted to the intensive care unit (ICU) in view of the acutely progressive weakness, with a preliminary diagnosis of demyelinating polyneuropathy (Guillain-Barré syndrome (GBS)).

The basic metabolic panel revealed hypokalemia, hypomagnesemia, and hypocalcemia. Besides this, he had elevated liver enzymes. An arterial blood gas revealed a pH of 7.615, pCO2 of 36.8 mmHg, and bicarbonate level of 38.3 mEq/L, indicating metabolic alkalosis. He had significantly elevated creatine phosphokinase (CPK) and lactate dehydrogenase (LDH). Myopathies such as polymyositis and rhabdomyolysis were considered. However, the rapid progression of clinical symptoms was suggestive of rhabdomyolysis. Finally, he had a low vitamin D level and low serum calcium. His initial laboratory values are summarized in Table 1.

**Table 1:** Laboratory profile of our patient on admission AST: aspartate transaminase, ALT: alanine transaminase, CPK: creatine phosphokinase, LDH: lactate dehydrogenase, TSH: thyroid-stimulating hormone

Laboratory test	Patient value	Reference range
Hemoglobin (g/dL)	12	13-17
Total leukocyte count (cells/cumm)	10,800	4,000-11,000
Platelet count (cells/cumm)	219,000	150,000-410,000
Urea (mg/dL)	19.1	16-40
Creatinine (mg/dL)	1.21	0.90-1.30
Sodium (mEq/L)	139.6	136-145
Potassium (mEq/L)	2.24	3.5-5.5
Calcium (mg/dL)	6.86	8.6-10
Chloride (mEq/L)	94.3	98-107
Magnesium (mg/dL)	1.3	1.6-2.6
Total bilirubin (mg/dL)	2.24	0.20-1.10
Direct bilirubin (mg/dL)	0.45	<0.20
AST (U/L)	351	19-48
ALT (U/L)	132	13-40
Albumin (g/dL)	3.95	2.8-4.4
Total protein (g/dL)	7.43	6.2-8.1
CPK (U/L)	24,774	20-200
LDH (U/L)	1,035	110-210
Random blood glucose (mg/dL)	84	74-106
TSH (IU/mL)	5.24	0.40-4.2
Vitamin D3 (ng/mL)	16.08	30-100

Routine electrocardiogram, echocardiogram, chest radiograph, and ultrasound of the abdomen were unremarkable. Serology for human immunodeficiency virus (HIV), hepatitis B virus (HBV), and hepatitis C virus (HCV) was performed to rule out infectious causes of polyneuropathy and was negative. Magnetic resonance imaging (MRI) of the brain and whole spine was performed to rule out central causes of quadriparesis and was unremarkable. Finally, nerve conduction studies (NCS) had findings for motor studies significant for reduced compound muscle action potential (CMAP) amplitudes of the left tibial and peroneal nerves from the surfaces of the left abductor hallucis and left extensor digitorum brevis muscles, respectively. Motor studies for the right tibial, right peroneal, bilateral median, and ulnar nerves were within normal limits. The sensory studies for the bilateral median, ulnar, superficial peroneal, and sural nerves were within normal limits. The F wave studies for the bilateral median, ulnar, peroneal, and tibial nerves were within normal limits. There were no findings suggestive of peripheral neuropathy.

To evaluate his hypokalemia, we sent for further investigations elaborated in Table 2, which pointed toward renal potassium wasting. His urine potassium was high at 18 mmol/g creatinine, and his spot urine potassium/creatinine (K/C) ratio was elevated at 1.8. His trans-tubular potassium gradient (TTKG), calculated as (urine potassium/serum potassium ) * (serum osmolarity/urine osmolarity), was 6, indicating an inappropriate renal response to hypokalemia. Finally, his plasma renin was suppressed, with normal aldosterone.

**Table 2:** Further laboratory investigations to confirm Gitelman syndrome

Laboratory test	Patient value	Reference range
Plasma renin (IU/mL)	<0.5	2.8-39.9
Serum aldosterone (ng/dL)	4.55	1.76-23.2
Calcium/creatinine molar ratio	0.034	<0.14
Urine potassium (mmol/L)	18	5-15
Urine chloride (mmol/L)	130	30-260
Spot urine creatinine (mg/dL)	42	22-328
Urine potassium/creatinine ratio	1.8	<1.5
Urine calcium	4.3	0.90-37.9

Based on the above laboratory findings, we narrowed our differential diagnoses down to either thiazide diuretic abuse or GS. Our patient’s denial of thiazide diuretic use led us to a final diagnosis of GS. Clinical exome sequencing for *SCL1A2*, *TRPM6*, and *CLCNKB* mutations was negative. Further, a detailed antibody panel for occult autoimmune disease was performed, which was negative. This included antinuclear antibody (ANA), anti-dsDNA, anti-nucleosome, anti-histone antibodies for systemic lupus erythematosus (SLE), anti-SS-A RO 60 KD, SS-A RO 52 KD, and SS-B LA antibodies for Sjögren’s syndrome, and anti-Scl 70 antibody and centromeric protein B (CENP-B) for systemic sclerosis. We also found negative results for antimitochondrial antibody (AMA) M2 for primary biliary cirrhosis (PBC) and anti-Jo1 for polymyositis and dermatomyositis. Anti-U1 ribonucleoproteins and antineutrophil cytoplasmic antibodies (ANCA) were also negative, leaving the root cause for our patient’s GS unclear.

With gradual correction of electrolyte abnormalities with intravenous infusion of potassium chloride, magnesium sulfate, and calcium gluconate with normal saline, there was rapid and significant clinical improvement, with the patient being able to walk independently within 48 hours. The patient was discharged with magnesium, calcium, and cholecalciferol tablets and potassium chloride syrup. The patient was also asked to follow up at regular intervals.

## Discussion

We report an uncommon cause of acute hypokalemic paralysis, GS. Clinically, the most commonly considered cause of acute, rapidly progressive weakness with global areflexia is GBS, an immune-mediated polyneuropathy [7,8]. Other relatively uncommon differential diagnoses include periodic paralysis, acute infectious polyneuropathies, and porphyric neuropathies [8].

In line with this, GBS was our initial clinical diagnosis. His lack of similar prior episodes of weakness made periodic paralysis unlikely, while his lack of fever made acute infectious polyneuropathy unlikely. This was further supported by the relatively symmetric, ascending progression of weakness, areflexia, and hypotonia on examination. However, the electrolyte abnormalities and lack of evidence of demyelination on NCS pointed us elsewhere. Urine electrolytes indicated renal potassium wasting, rather than hypokalemia due to poor intake or extrarenal losses. The lack of hypertension ruled out hyperaldosteronism as a potential cause, later confirmed by investigations, narrowing the differential diagnosis to diuretic abuse or renal tubulopathy, either GS or Bartter syndrome (BS). BS is typically more severe, manifesting in early childhood, with normal magnesium levels [9], pointing us to GS.

GS is a rare renal tubulopathy characterized by hypokalemia, hypomagnesemia, hypocalciuria, and metabolic alkalosis. It occurs due to the disruption of sodium and chloride reabsorption in the distal convoluted tubule (DCT). The resulting volume contraction results in renin-angiotensin-aldosterone system (RAAS) axis activation, promoting sodium reabsorption in the collecting tubules. This reabsorption occurs in exchange for potassium and hydrogen ions, resulting in hypokalemia and metabolic alkalosis [6]. Further, the lack of sodium reabsorption in the DCT causes membrane hyperpolarization, resulting in hypocalciuria [6]. The mechanism of hypomagnesemia is not well understood. Mutations in *TRPM6*, an Mg2+-permeable channel along the apical membrane of the distal convoluted tubule (DCT), have been reported to cause increased magnesium excretion and reduced intestinal magnesium reabsorption [10].

GS can be inherited or acquired. Inherited GS is most commonly due to mutations in the *SLC12A3* gene that encodes the thiazide-sensitive sodium-chloride cotransporter, with other implicated genes being *TRPM6*, *HNF1B*, *CLCNKB*, and mitochondrial genes [11]. It typically presents in adolescence or early adulthood [12]. Acquired GS presents later; is usually secondary to autoimmune diseases, including Sjögren’s syndrome, systemic lupus erythematosus, and autoimmune thyroiditis, among others; and lacks the typical genetic mutations [12]. Given our patient’s age, we suspected that acquired GS was more likely. However, both clinical exome sequencing for *SCL1A2*, *TRPM6*, and *CLCNKB* mutations and detailed autoimmune panel were both negative. Given that most literature regarding the genetic spectrum of GS originates from outside South Asia [1,11], it is possible that his GS was due to a hitherto unreported, relatively benign mutation. It is also possible that it was secondary to a seronegative autoimmune process.

Most commonly, GS is asymptomatic or has mild symptoms such as fatigue, myalgia, muscle cramps, salt craving, and excessive thirst [1]. GS causing acute hypokalemic paralysis that too as the initial manifestation is extremely rare, with only occasional reports [2-6]. An additional anomaly of our patient’s presentation is the presence of rhabdomyolysis. We hypothesize that this was hypokalemic rhabdomyolysis secondary to GS [13,14]. Potassium is required for regional vasodilation during muscle activity [13,15]. Hypokalemia can impair this, causing relative ischemia of the muscle [13]. Our patient had hypocalcemic tetany. The muscle activity, combined with the severity of hypokalemia, may have led to muscle ischemia and rhabdomyolysis.

The management of GS is uncomplicated and involves the gradual correction of electrolyte abnormalities. Our patient was managed with an intravenous infusion of potassium chloride, magnesium sulfate, and calcium gluconate with normal saline, which led to a rapid reversal of symptoms.

## Conclusions

We report the rare initial manifestation of GS as acute hypokalemic paralysis, leading to a diagnostic dilemma. We have also highlighted the similarities in the presentation of GBS and hypokalemic paralysis. It is important to distinguish between them as their therapeutic interventions differ significantly. GS is a rare renal salt wasting disease, whose management hinges on gradual correction of dyselectrolytemia. More literature from the Indian subcontinent regarding the clinical and genetic spectrum of GS is needed.
